# Retracted: Inflammatory Microenvironment Promotes Hemopoietic-Derived Angiogenic Cell Expansion and Arterial Specification

**DOI:** 10.1155/2018/4082363

**Published:** 2018-08-09

**Authors:** The Scientific World Journal

The Scientific World Journal and the authors have retracted the article titled “Inflammatory Microenvironment Promotes Hemopoietic-Derived Angiogenic Cell Expansion and Arterial Specification” [1]. The article was found to contain duplicated images from the authors' previous article in Blood, Zeoli et al. [2], after concerns were raised on PubPeer. Details of the duplication are as follows:

(1) pSTAT in Figure 7(d) in Blood and STAT in Figure 1 in The Scientific World Journal are the same, representing phosphorylated and unphosphorylated protein, respectively, and the *β*-actin panels are the same.

(2) Figure 1(Aa) in The Scientific World Journal is modified from Figure 4(Ci) in Blood, where the top of each image is the same, but parts of the top right of Figure 4(Ci) are duplicated in the bottom left of Figure 1(Aa).

(3) The lower panels of Figure 4(b) in Blood, when rotated left, are the same as the panels in Figure 1(c) in The Scientific World Journal. The middle panel in Blood is the same as that in The Scientific World Journal when the contrast and exposure are adjusted, representing Eph4 and EphrinB2, respectively.

The corresponding author Dr. Brizzi apologizes for this.

## References

[1] Dentelli P., Brizzi M. F. (2008). Inflammatory microenvironment promotes hemopoietic-derived angiogenic cell expansion and arterial specification. *The Scientific World Journal*.

[2] Zeoli A., Dentelli P., Rosso A. (2008). Interleukin-3 promotes expansion of hemopoietic-derived CD45+ angiogenic cells and their arterial commitment via STAT5 activation. *Blood*.

